# Grandchild’s IQ is associated with grandparental environments prior to the birth of the parents

**DOI:** 10.12688/wellcomeopenres.16205.2

**Published:** 2021-03-15

**Authors:** Jean Golding, Gerard van den Berg, Kate Northstone, Matthew Suderman, Genette Ellis, Yasmin Iles-Caven, Steve Gregory, Marcus Pembrey

**Affiliations:** 1 Population Health Sciences, Bristol Medical School, University of Bristol, Bristol, BS8 2BN, UK; 2 Department of Economics, University of Bristol, Bristol, BS8 2BN, UK

**Keywords:** ALSPAC, IQ, Cognition, Transgenerational effects, Grandmaternal smoking, Grandfather, GDP

## Abstract

*Background*. Despite convincing animal experiments demonstrating the potential for environmental exposures in one generation to have demonstrable effects generations later, there have been few relevant human studies. Those that have been undertaken have demonstrated associations, for example, between exposures such as nutrition and cigarette smoking in the grandparental generation and outcomes in grandchildren. We hypothesised that such transgenerational associations might be associated with the IQ of the grandchild, and that it would be likely that there would be differences in results between the sexes of the grandparents, parents, and children.

*Method. *We used three-generational data from the Avon Longitudinal Study of Parents and Children (ALSPAC).  We incorporated environmental factors concerning grandparents (F0) and focussed on three exposures that we hypothesised may have independent transgenerational associations with the IQ of the grandchildren (F2): (i) UK Gross Domestic Product (GDP) at grandparental birth year; (ii) whether grandfather smoked; and (iii) whether the grandmother smoked in the relevant pregnancy. Potential confounders were ages of grandparents when the relevant parent was born, ethnic background, education level and social class of each grandparent.

*Results*. After adjustment, all three target exposures had specific associations with measures of IQ in the grandchild. Paternal grandfather smoking was associated with reduced total IQ at 15 years; maternal grandfather smoking with reduced performance IQ at 8 years and reduced total IQ at 15.  Paternal grandmother smoking in pregnancy was associated with reduced performance IQ at 8, especially in grandsons. GDP at grandparents’ birth produced independent associations of reduced IQ with higher GDP; this was particularly true of paternal grandmothers.

*Conclusions. *These results are complex and need to be tested in other datasets. They highlight the need to consider possible transgenerational associations in studying developmental variation in populations.

## Introduction

Studies of IQ variation in populations have revealed several unexplained phenomena. Twin studies of IQ show greater correlation in monozygotic than same-sex dizygotic twins resulting in heritability estimates ranging from 0.5 to 0.8 ( Plomin & Deary, 2015), with estimates rising throughout early development, reaching around 0.8 at 18–20 years and continuing at that level well into adulthood. ( Bouchard, 2013). This heritability is generally assumed to be genetic – large numbers of genetic variants each with tiny effects – but to date genome wide association studies (GWAS) explain only a relatively small proportion of the variance in IQ. A recent, large-scale GWAS meta-analyses, that combined IQ and educational attainment data, found that common SNPs were only able to account for roughly half of the overall heritability of the phenotype ( Lam *et al.*, 2017). As with the heritability of height and other complex traits ( Boyle *et al.*, 2017; Yang *et al.*, 2015) there is a tendency to assume that any missing heritability in IQ is due to many, as yet undiscovered, DNA sequence variants. Whilst this is plausible, the possible contribution of environmental exposures both directly and indirectly through non-genetic (or epigenetic) biological inheritance should not be ignored. There is consistent evidence in several industrialised countries that the IQ of the population has risen over the decades, known as the Flynn effect ( Dickens & Flynn, 2001; Pietschnig & Voracek, 2015; Trahan *et al.*, 2014). A meta-analysis of studies published by 1997 also found evidence supporting the hypothesis that there was a strong intrauterine effect that accounted for some of the heritability estimates ( Devlin *et al.*, 1997). There is evidence that aspects of diet, such as prenatal diet of the mother and whether the infant was breast fed, also have strong associations with IQ in the child ( Hibbeln *et al.*, 2007; Kramer *et al.*, 2008).

Furthermore, exposures need not be direct to have an influence. There is increasing evidence that intrauterine environments may affect not only the developing fetus but also the subsequent generation. Using grandmaternal smoking in pregnancy as the exposure, we have demonstrated this for grandchild’s growth, particularly in adolescence ( Golding *et al.*, 2014). As part of these initial intergenerational anthropometric studies using data from the Avon Longitudinal Study of Parents and Children (ALSPAC), we showed that boys with both mothers and paternal grandmothers who had smoked in pregnancy had significantly smaller heads at birth than their peers whose mothers had smoked but whose paternal grandmothers had not smoked. This intriguing finding was robust to the effects of confounding by current lifestyle and socio-economic background ( Pembrey *et al.*, 2014). Knowing the evidence that a small head circumference at birth is associated with a lower IQ later in childhood ( Gale *et al.*, 2006), we demonstrated that these particular boys had lower IQ scores on average, and that this was particularly due to the verbal component ( Pembrey *et al.*, 2014). We should emphasise, however, that this was part of a study focused on anthropometric outcomes, and we have recently begun to investigate this question in terms of a range of neurological and behavioural outcomes. We have so far uncovered associations between maternal grandmother smoking prenatally and autistic traits in the grandchild ( Golding *et al.*, 2017), and between paternal grandmother smoking and early onset myopia in the grandchild ( Williams *et al.*, 2019). The present study is focused on the grandparental environment prior to the birth of the parents and cognition in the grandchildren in childhood and late adolescence. The currently available data on grandparental exposures include grandfathers’ smoking, the GDP of the year of birth of each grandparent (both the trend and the business cycle), in addition to grandmaternal smoking during the pregnancy resulting in the birth of the parent, where tentative transgenerational associations with the grandchild’s IQ have already been observed. We hypothesise that grandfathers’ smoking may show transgenerational IQ associations, since smoking by fathers in mid-childhood is associated with altered growth and development of their offspring into adulthood ( Golding *et al.*, 2019; Northstone *et al.*, 2014). The GDP of the year of the birth of the grandparents is a potential candidate for transgenerational associations, given recent population findings of associations between this factor and both newborn health ( van den Berg *et al.*, 2020) and cardiovascular health in adulthood ( Alessie *et al.*, 2019).

Most inter/transgenerational associations can be expected to have contributions from social patterning, genetic (DNA sequence) inheritance and non-genetic gametic inheritance, such as transmission of epigenetic information to the next generation(s). Understanding the contribution of each to the variation in cognitive ability will be an immense challenge, needing comprehensive, multigenerational, prospective cohort studies. The current study represents a start. For the purposes of this paper, which does not include molecular/epigenetic analyses, we will just use ‘transgenerational’ as a general term for the observed associations between grandparental exposures and grandchild’s outcomes, irrespective of whether direct exposure of the germline destined for the grandchild may have occurred. 

## Methods

### The study sample

ALSPAC is a pre-birth cohort designed to determine the environmental and genetic factors that are associated with health and development of the study offspring ( Boyd *et al.*, 2013; Fraser *et al.*, 2013; Golding *et al.*, 2001). It recruited pregnant women who were resident in Avon, UK with expected dates of delivery between 1st April 1991 and 31st December 1992 (an estimated 80% of the eligible population). The initial number of pregnancies enrolled was 14,541 (for these at least one questionnaire had been returned or a “Children in Focus” clinic had been attended by 19/07/99). Of these initial pregnancies, there was a total of 14,676 fetuses, resulting in 14,062 live births and 13,988 children who were alive at 1 year of age. Data were collected at various time-points using self-completion questionnaires, biological samples, hands-on measurements, and linkage to other data sets. Full details of all the data collected are available on the study website: www.bristol.ac.uk/alspac/researchers/data-access/data-dictionary/. 

As part of the study design, there was a concerted effort before the child’s birth to obtain from each of the parents (F1) details of their own parents (F0). The pregnant women were sent four questionnaires during pregnancy, one of which requested details of their parents; in parallel they were sent two questionnaires for their partners to complete, one of which included similar questions on the partner’s own parents.

### Ethical approval

Ethical approval for the study was obtained from the ALSPAC Ethics and Law Committee (ALEC) (ALEC IRB00003312) (registered on the Office of Human Research Protections database as UBristol IRB#1) and the three NHS Local Research Ethics Committees (LRECs) that covered the study area (Southmead, Bristol & Weston and Frenchay). ALEC agreed that consent was implied if questionnaires were returned ( Birmingham, 2018). Further detailed information on the ways in which confidentiality of the cohort is maintained and a full list of ethical approvals may be found on the study website: http://www.bristol.ac.uk/alspac/researchers/research-ethics/

### The outcome measures: IQ tested at ages 8 and 15

The study children (F2) were invited to attend for a half day of tests when they were aged 8. The tests included the WISC-III ^UK ^( Wechsler *et al.*, 1992) to assess cognitive function. At the time it was the most up to date version of the Weschler Intelligence Scale for Children, the most widely used individual ability test worldwide. We used a short form of the measure with alternate items (always starting with item number 1 in the standard form) for all subtests, except for the coding subtest which was administered in its full form. This resulted in a reduction in the length of the session and the children were less likely to tire; such reduced formats of the WISC have been used successfully in several studies (e.g. Finch & Childress, 1975; Stricker *et al.*, 1968). The tests were administered by members of the ALSPAC psychology team and the WISC IQ scores (verbal, performance and total IQ) were calculated from the total scaled scores using the look-up tables provided in the WISC manual.

When the study children were aged 15, the Wechsler Abbreviated Scale of Intelligence, Second Edition (WASI-II) ( McCrimmon & Smith, 2013; Wechsler, 2011) was administered. This is an updated abbreviated measure of cognitive intelligence designed for individuals 6 to 90 years of age. It was developed to quickly and accurately estimate cognitive intelligence when administration of a full battery was not feasible or necessary. The full WASI-II consists of four subtests, selected as those with the highest factor loadings on *g*, the estimate of general intelligence. Because of time and cost restraints just two subtests were administered in ALSPAC: the vocabulary and matrix reasoning subtests, which were combined to form the Full-Scale IQ-2 subtest.

### The exposures

The questionnaires sent to the parents (F1) during pregnancy elicited information on the following items of relevance to this project:

(i) The smoking histories of each of their own parents (i.e. the study grandparents (F0)).
(ii) Parents were asked: ‘Did your mother ever smoke?’ If the response was ‘yes’, they were asked: ‘Did she smoke when she was expecting you?’ and given the option responses ‘yes / no/ don’t know’. Thus, the parents who replied ’don’t know’, had a mother (F0) who smoked but the parent (F1) was unsure whether she had smoked during her pregnancy. As with our other studies, we have analysed these data assuming that these women did smoke during pregnancy ( Golding *et al.*, 2014; Miller *et al.*, 2014a; Miller *et al.*, 2014b; Pembrey *et al.*, 2014). This assumption was shown to have face validity by demonstrating that the mean birthweight of this group of study mothers was reduced when compared with those who reported that their mother had definitely not smoked in pregnancy ( Pembrey *et al.*, 2014).
(iii) The ages of each grandparent (F0) when the study parent (F1) was born.
(iv) For each of the four study grandparents (F0) their years of birth were estimated from details of their ages at the time of birth of the relevant parent and the age of the parent when the study child was born.
(v) Two variables are based on the annual real Gross Domestic Product per capita (GDP) at the time of the birth of each grandparent. This is based on the total amount produced in the UK in a year, per inhabitant, and corrected for inflation. “Corrected for inflation” here means that everything is expressed in terms of 1990 US dollars. The annual real GDP per capita comes from Maddison (2003); Maddison, 2013 (updated) who provided internationally comparable historical macro-economic time series of such variables. Somewhat loosely, the annual real GDP per capita indicates the average economic activity per inhabitant in each year. We have used both the annual real GDP decomposed into a trend and a business cycle. The trend captures long-run trends in economic activity. The cycle is the business cycle fluctuating along the trend. The decomposition is the Hodrick-Prescott filter with smoothing parameter 100, over the years 1835–2001. The sum of trend and cycle equals the original variable.
(vi) The social class of each grandparent (based on their occupations as reported by their offspring, the study parents (F1).
(vii) The educational qualifications of each grandparent (grouped into the equivalent of O-level or higher, and lower than this).
(viii) Ethnic group (grouped as white and all other).
(ix) Parity (for grandmothers only) – i.e. whether the study parent was the first or later birth to that grandmother.

These variables were considered as possible inter/trans-generational candidates and/or possible confounders ( Table 1).

**Table 1.** Characteristics of the study grandparents (F0) according to their likely categorisation.

Measure of grandparents	MGM	MGF	PGM	PGF
Year of birth	S	S	S	S
GDP at year of birth	I	T	T	T
Business cycle at year of birth	I	T	T	T
Age at birth of parent	S+I	S+T	S+T	S+T
Ethnic group	S+G	S+G	S+G	S+G
Education level	S+G	S+G	S+G	S+G
Social class	S	S	S	S
Parity of grandmother at birth of parent	S+I	-	S+T	-
Grandmother smoked in pregnancy resulting in parent	I	-	T	-
Ever smoked	I	T	T	T

Exposures are categorised according to whether the associations with grandchild’s (F2) IQ are likely to be transgenerational (T), intergenerational (I), genetic (G) or socially transmitted (S). MGM = maternal grandmother; MGF = maternal grandfather; PGM = paternal grandmother; PGF = paternal grandfather.

**Table 2.** The data available for analysis.

Measure Available	Grandparent	N	% (n) IQ at 8	% (n) IQ at 15
Year of birth				
	MGM	10925	57.4 (6268)	41.4 (4523)
	MGF	8745	67.3 (5889)	49.0 (4282)
	PGM	8084	58.1 (4696)	42.9 (3468)
	PGF	7693	58.8 (4523)	43.3 (3334)
Ethnic origin				
	MGM	12066	55.7 (6717)	40.0 (4824)
	MGF	12018	55.7 (6694)	40.0 (4803)
	PGM	9679	56.1 (5427)	40.9 (3958)
	PGF	9646	56.1 (5412)	41.0 (3952)
Education level				
	MGM	9252	55.4 (5129)	40.7 (3763)
	MGF	8810	55.0 (4844)	40.2 (3542)
	PGM	7505	56.0 (4200)	40.7 (3054)
	PGF	7544	55.9 (4214)	40.7 (3069)
Smoked – ever				
	MGM	12677	52.1 (6605)	37.3 (4728)
	MGF	12449	52.4 (6526)	37.5 (4674)
	PGM	9646	56.2 (5417)	40.9 (3945)
	PGF	8921	56.3 (5024)	41.2 (3671)
Parity				
	MGM	12432	54.7 (6798)	39.2 (4879)
	PGM	3688	56.1 (2070)	43.0 (1587)
Smoked in pregnancy				
	MGM	12620	52.1 (6576)	37.3 (4705)
	PGM	9602	56.2 (5393)	40.9 (3930)

MGM = maternal grandmother; MGF = maternal grandfather; PGM = paternal grandmother; PGF = paternal grandfather. N denotes the number of the original population of parents who completed questions on their own parents. the proportions whose study child had a valid IQ measure are shown in columns 4 and 5.

### Statistical approach

The analyses are designed to determine the relationships between different aspects of the environment of the grandparents (F0) of the study children (F2), particularly those that might indicate an inter- or trans-generational association (see Table 1). Among those that might indicate such a mechanism, we highlight: (a) the grandmother who smoked cigarettes during the pregnancy that resulted in the birth of the study parent (F1); (b) the study grandfather who smoked (we have assumed that all such smokers will have started smoking before the study parent was conceived); (c) the trend level in the GDP of the year of birth of each grandparent; and (d) the business cycle at the year of birth of each grandparent (independent of the trend). The variables that are more likely to be confounders were the education levels, ethnic group, social class and year of birth of each grandparent. We were agnostic about assignment of grandparental ages and parity of the grandmother at the birth of the parent to just one of these two categories of variables as they could be considered to be in either category.

We analysed the data in a hypothesis free structure, taking care to ensure that we avoided Type I errors as much as possible; consequently, we did not allow for multiple testing. However, based on previous results from the animal and human literature, we hypothesised that if there were effects of an inter/trans-generational nature, we anticipated that the associations would differ between the sexes. We therefore analysed the grandsons and granddaughters separately for each of the maternal and paternal inheritance lines. 

The IQ score of the child was analysed as a continuous variable (using multiple regression). The initial analyses were unadjusted. In order to take account of the collinearity between many of the factors considered we employed a backwards step-wise approach using all grandparental variables that were associated with the unadjusted outcome at P<0.05 to determine the factors independently associated with the grandchild’s IQ level. The analyses were undertaken for each parent (F1), and for each sex of the F2 generation. Because this was a hypothesis-generating study we did not apply any correction for multiple testing. Nor did we use features of the parents as confounders. Instead, the social and educational features of the grandparents were used in the stepwise regression as confounders (which is essentially controlling for the socio-economic features of the parents’ childhoods). To then take into account other aspects of the parents and/or of the children as confounders would likely identify mechanisms by which the associations between exposures to the grandparents and the child’s IQ may have taken place, and could nullify the transgenerational effects themselves. Determining the possible mechanisms by which any transgenerational effects occur will be an important exercise for the future. 

In order to clarify the methodology, we first describe in detail the results for the Total IQ at age 8, so that the reader may understand the logical sequence of analyses. Thereafter we summarise the results in the text for other outcomes and illustrate the results in *Extended data*, Tables S1–S28b ( Iles Caven *et al*., 2020).

## Results

### Total IQ at age 8

The numbers of parents who originally answered the questions on their own parents is shown in Table 2. These were answered in self-completion questionnaires when the mother was pregnant – by definition, she was resident in the study area at the time. In general, the study fathers were less likely to have answered these questions than the study mothers.

The first 8 years of the study resulted in many household moves of the study families, including within Avon, within the United Kingdom and to the rest of the world. The study children (F2) who did not attend to have their IQ measured included: those who were living outside the study area, those whose parents decided that the visit would not be appropriate for their child (e.g. children with autism), those who were lost to follow up or who had died. For children (F2) whose parents (F1) had answered the questions on their own parents (F0) the proportion who had IQ scores measured was approximately 55% at age 8, and 40% at age 15. IQ measures were less likely to have been obtained when the parents (F1) were younger and/or had lower levels of education or were resident in public housing (data not shown). 

***Relationships between grandchild’s mean Total IQ at 8 and grandparental characteristics*.** The ways in which the mean IQ of the grandchildren varied with characteristics of their four grandparents is shown in Table 3. These unadjusted data show that the maternal grandparents were more likely (at P<0.01) to have grandchildren with significantly higher mean IQ if they: (a) were born before the Second World War (1940), and especially if before 1930; (b) were born when the GDP level was low; (c) had an educational qualification of at least O-level standard; (d) gave birth to the study parent at age 25 or older; (e) were of a relatively high social standing; (f) were non-smokers; and if (g) the maternal grandmother had not smoked when expecting the study parent (F1). There was a marginal decrease in grandchild’s IQ if the maternal grandmother (but no other grandparents) was non-white. Information on the grandmother’s parity at the birth of the study mother was available in a reduced sample; there was an association if the study father was the paternal grandmother’s first-born child.

**Table 3.** Mean IQ of grandchildren at age 8 for information on the grandparents. When more than 2 categories are given P-values are given for trend; associations when P<0.05 are highlighted in bold; and potential transgenerational associations are printed in red).

	MGM	MGF	PGM	PGF
Year of birth				
<1920	**108.8**	**108.7**	**107.7**	**108.3**
1920-4		**107.7**		**107.3**
1925-9	**107.4**	**107.2**	**107.9**	**108.3**
1930-4	**106.3**	**105.9**	**108.2**	**107.8**
1935-9	**104.9**	**104.0**	**106.2**	**104.6**
1940-4	**103.0**	**101.9**	**102.7**	**103.0**
1945+	**99.0**	**98.8**	**99.7**	**98.9**
Total N	**6268 ^a^**	**5889 ^a^**	**3732 ^a^**	**3595 ^a^**
				
Year of birth ^w^	**-3.04 ^a^**	**-2.57 ^a^**	**-2.05 ^a^**	**-1.80 ^a^**
				
Business cycle at birth ^x^	**-2.81 ^a^**	**-2.68 ^a^**	**-2.23 ^a^**	**-2.15 ^a^**
				
GDP at birth ^y^	-0.10	-0.08	-0.29	0.00
				
Ethnic group				
White	**104.5**	104.5	105.3	105.4
Non-white	**101.2**	102.9	103.8	104.4
Total N	**6717 ^d^**	6694	5427	5412
				
Education level				
≥O-level	**108.9**	**109.3**	**110.3**	**109.5**
<O-level	**103.4**	**102.6**	**104.0**	**104.0**
Total N	**5129 ^a^**	**4844 ^a^**	**4200 ^a^**	**4214 ^a^**
				
Ever smoked				
Yes	**103.6**	**103.7**	**104.3**	**105.0**
No	**105.3**	**106.3**	**106.7**	**107.2**
Total N	**6605 ^a^**	**6526 ^a^**	**5417 ^a^**	**5024 ^b^**
				
Age at birth of parent (F1)				
<25	**102.5**	**102.5**	**103.9**	**103.5**
25–34	**106.1**	**105.4**	**107.1**	**106.8**
35–39	**106.4**	**107.1**	**106.9**	**107.1**
40+		**106.0**		**107.1**
Total N	**6268 ^a^**	**5889 ^a^**	**4696 ^a^**	**4518 ^a^**
				
Trend with age ^z^	**+1.81 ^a^**	**+1.30 ^a^**	**+1.32 ^a^**	**+1.11 ^a^**
				
Social class				
I	**107.7**	**111.2**	**114.4**	**111.4**
II	**108.7**	**108.4**	**108.3**	**108.5**
IIINm	**105.2**	**106.4**	**106.0**	**107.8**
IIIM	**101.4**	**101.8**	**103.5**	**103.0**
IV	**102.3**	**102.9**	**103.8**	**103.2**
V	**100.0**	** 98.4**	**100.7**	**102.5**
Total N	**3791 ^az^**	**5597 ^az^**	**2865 ^az^**	**5033 ^az^**
				
Parity at birth of parent				
0	102.9		**108.2**	
1+	105.1		**105.5**	
Total N	6798		**2070 ^b^**	
Grandmother smoked in pregnancy				
Yes	**102.9**	**-**	**103.9**	-
No	**105.1**	**-**	**106.4**	-
Total N	**6576 ^a^**	**-**	**5393 ^a^**	-
				

MGM = maternal grandmother; MGF = maternal grandfather; PGM = paternal grandmother; PGF = paternal grandfather. ^a^P<0.0001; ^b^P<0.001; ^d^P<0.05. ^w^trend per 5-year grouping; ^x^trend standardised GDP per year; ^y^Trend per year of birth; ^z^trend per category above.

These associations are put in context when the R ^2^ values (contribution to the variance) from each variable are considered ( Table 4). This shows that, when using the unadjusted comparisons, the major contributor to the variance of the grandchild’s IQ level are the grandparents’ years of birth, the GDP trend level at the time of their birth, their education levels and their social classes.

**Table 4.** Summary of unadjusted associations (R ^2^%) between grandparental background features and their grandchild’s 8-year-old total IQ. Potential transgenerational associations are printed in red.

Measure concerning Grandparent (F0)	MGM		MGF		PGM		PGF	
	n	R ^2^%	n	R ^2^%	n	R ^2^%	N	R ^2^%
Year of birth	6268	3.14 ^a^	5889	2.26 ^a^	3732	1.45 ^a^	3595	1.13 ^a^
GDP at birth	6268	2.83 ^a^	5889	2.45 ^a^	3732	1.70 ^a^	3595	1.54 ^a^
Business cycle	6268	0.00	5889	0.00	3732	0.03	3595	0.00
Ethnic origin	6717	0.06 ^d^	6694	0.02	5427	0.02	5412	0.01
Education level	5129	2.57 ^a^	4844	3.88 ^a^	4200	3.24 ^a^	4214	2.64 ^a^
Smoked	6605	0.26 ^b^	6526	0.47 ^b^	5417	0.53 ^a^	5024	0.30 ^a^
Age at birth of parent (F1)	6268	1.19 ^a^	5889	0.61 ^a^	4696	0.64 ^a^	4518	0.44 ^a^
Social class	3791	2.83 ^a^	5597	4.27 ^a^	2863	2.21 ^a^	5033	3.03 ^a^
Parity	6798	0.00	-	-	2070	0.60 ^b^	-	-
Smoked when expecting parent	6576	0.39 ^a^	-	-	5393	0.55 ^a^	-	-

MGM = maternal grandmother; MGF = maternal grandfather; PGM = paternal grandmother; PGF = paternal grandfather. ^a^P<0.0001; ^b^P<0.001; ^d^P<0.05.

***Transgenerational associations: Total IQ at 8*.** Contribution to the variance is not equivalent to effect sizes and does not necessarily indicate which factors are independent of one another. We therefore first carried out backwards step-wise multiple regression using the variables in the **maternal line**. Dropped from the final model were the following potential transgenerational associations: the GDP trend level associated with the maternal grandmother’s year of birth, whether the maternal grandfather was a smoker, whether the grandmother smoked in the pregnancy resulting in the birth of the study mother. The independent factors associated with the grandchild’s IQ were all features where the transgenerational associations were likely to be learnt rather than inherited ( Table 5).

**Table 5.** Results of stepwise analysis of grandchild’s total IQ at 8 involving the maternal line. β is the standardised regression coefficient with 95% confidence interval; potential transgenerational associations are printed in red.

	Unadjusted analyses			Adjusted analysis	
Maternal line	N	β [95% CI]	P	b [95% CI]	P
Maternal grandmother’s year of birth ^x^	6267	-0.36 [-0.41. -0.31]	**1.3 x 10 ^-45^**	-0.53 [-0.64, -0.41]	**6.4 x 10 ^-19^**
GDP in MGM’s birth year ^y^	6267	-2.89 [-3.30, -2.47]	**1.5 x 10 ^-42^**		
Mother’s ethnicity: white v non-white	6716	3.30 [0.15, 6.46]	**0.040**		
Maternal grandmother’s education level	5128	2.59 [2.25, 2.94]	**1.1 x 10 ^-48^**	1.33 [0.84, 1.81]	**8.3 x 10 ^-8^**
Maternal grandfather’s education level	4843	2.70 [2.38, 3.01]	**1.4 x 10 ^-60^**	1.07 [0.60, 1.54]	**8.1 x 10 ^-6^**
Maternal grandfather ever smoked	6525	-2.59 [-3.50, -1.68]	**2.8 x 10 ^-8^**		
Grandmother’s age at birth of mother	6267	0.30 [0.24, 0.38]	**4.7 x 10 ^-18^**	-0.33 [-0.49, -0.16]	**1.3 x 10 ^-4^**
Grandfather’s age at birth of mother	5888	0.19 [0.13, 0.26]	**2.2 x 10 ^-9^**		
MGM parity at birth of mother	6797	-0.13 [-0.96, 0.71]	0.763		
Maternal grandfather’s social class	5596	-2.76 [-3.11, -2.42]	**4.5 x 10 ^-55^**	-1.33 [-1.82, -0.85]	**6.1 x 10 ^-8^**
MGM smoked when pregnant with mother	6576	-2.15 [-2.99, -1.32]	**< 0.0001**		

^x^Trend in IQ per year of grandparent’s birth; ^y^trend in IQ per standardised GDP per year; MGM = maternal grandmother. N = 3670; R ^2^ = 9.85%

The pattern of association between the characteristics of the paternal grandparents and the grandchild’s IQ was similar to that shown for the maternal grandparents, with positive associations if the grandparents were born prior to the Second World War; were more educated; were non-smokers; aged 25 at least at the birth of the study father; belonged to a non-manual social class based on their occupations. In addition, there was a negative association when the paternal grandmother smoked in the pregnancy resulting in the birth of the study father ( Table 3). We used a similar analysis for the **paternal line** as was carried out for the maternal line. Of the highly significant unadjusted associations, only one of the presumed transgenerational associations remained in the adjusted analysis: the GDP trend level in the paternal grandmothers’ year of birth ( Table 6). The adjusted result: β = -1.86; 95% CI -2.50, -1.22; P = 1.2 × 10 ^-8^ suggests that the lower the GDP trend level at the paternal grandmothers’ birth, the higher the IQ level of her grandchild.

**Table 6.** Results of stepwise analysis of grandchild’s total IQ at 8 involving the paternal line. β is the standardised regression coefficient with 95% confidence interval; potential transgenerational associations are printed in red.

	Unadjusted analyses			Adjusted analysis	
PATERNAL LINE	N	β [95% CI]	P	β [95% CI]	P
Paternal grandmother’s year of birth ^x^	3736	-0.23 [-0.29, -0.17]	**1.7 x 10 ^-13^**		
Paternal grandfather’s year of birth ^x^	3599	-0.19 [-0.25, -0.13]	**1.3 x 10 ^-10^**		
GDP in PGM’s birth year ^y^	3736	-2.31 [-2.86, -1.76]	**1.5 x 10 ^-16^**	-1.86 [-2.50, -1.22]	**1.2 x 10 ^-8^**
Paternal grandmother’s education level	4200	2.54 [2.18, 2.91]	**5.1 x 10 ^-41^**	2.18 [1.69, 2.66]	**2.8 x 10 ^-18^**
Paternal grandfather’s education level	4214	2.07 [1.73, 2.40]	**1.4 x 10 ^-32^**		
Grandmother’s age at birth of father	4696	0.22 [0.14, 0.30]	**4.1 x 10 ^-8^**		
Grandfather’s age at birth of father	4518	0.16 [0.09, 0.23]	**7.4 x 10 ^-6^**		
Paternal grandfather’s social class	5033	-2.37 [-2.74, -2.00]	**1.4 x 10 ^-35^**	-1.05 [-1.59, -0.52]	**1.3 x 10 ^-4^**
PGM smoked when pregnant with father	5393	-2.47 [-3.36, -1.58]	**<0.0001**		

^x^Trend in IQ per year of grandparent’s birth; ^y^trend in IQ per standardised GDP per year; PGM = paternal grandmother. N=2708; R ^2^=6.10%

***Differences in results between the sexes of the grandchildren*.** Comparison of the separate analyses of the **maternal** grandparents according to the sex of the grandchild showed that no potential transgenerational association was retained in either model ( *Extended data*, Tables S1 and S2) ( Iles Caven *et al*., 2020). However, for the **paternal** line, as with the sexes combined, there were associations with the trend in GDP with the year of birth of the paternal grandmother for both the grandsons and granddaughters. There were major differences in effect sizes between the sexes in the adjusted trend in GDP of the paternal grandmother’s birth year. For both the grandsons and granddaughters, the adjusted regression coefficient had a P value < 4×10 ^-4^, but the association relating to the granddaughters was significantly more negative than that for the grandsons (P for interaction < 0.05) ( Table 7 and *Extended data*, Tables S3 and S4) ( Iles Caven *et al*., 2020).

**Table 7.** Summary of all adjusted results with potential transgenerational associations associated with 8-year-old grandchild’s total IQ. See *Extended data*, Tables S1–S27 ( Iles Caven *et al*., 2020) for details of analyses.

Exposure to Grandparent	F0	Outcome	Sex of Grandchild	β [95% CI]
GDP of birth year ^y^	PGM	Total IQ at 8	All	-1.86 [-2.50, -1.22] ^a^
			Boys	-1.74 [-2.70, -0.78] ^a^
			Girls	-3.08 [-4.34, -1.82] ^a^
	PGF	Total IQ at 15	All	-2.99 [-3.82, -2.15] ^a^
			Girls	-2.28 [-3.12, -1.44] ^a^
	PGM	Verbal IQ at 8	All	-2.81 [-3.77, -1.85] ^a^
			Boys	-2.04 [-2.98, -1.09] ^a^
			Girls	-3.10 [-4.41, -1.80] ^a^
	PGM	Performance IQ	Girls	-2.74 [-4.05, -1.43] ^a^
	MGM	Total IQ at 15	All	-2.01 [-3.68, -0.33] ^d^
			Boys	-4.73 [-5.90, -3.55] ^a^
	MGF	Performance IQ	All	-2.00 [-2.61, -1.38] ^a^
	MGM		Boys	-1.80 [-2.64, -0.97] ^a^
GDP business cycle ^z^	PGM	Verbal IQ at 8	Girls	+0.90 [+0.04, +1.75] ^d^
Grandfather smoked	PGF	Total IQ at 15	All	-2.75 [-4.40, -1.09] ^c^
			Girls	-2.53 [-4.32, -0.73] ^b^
	MGF	Total IQ at 15	All	-1.07 [-2.13, -0.02] ^d^
			Girls	-1.61 [-2.98, -0.24] ^d^
		Performance IQ	All	-2.03 [-3.30, -0.76] ^b^
			Boys	-2.92 [-4.69, -1.14] ^c^
			Girls	-1.87 [-3.50, -0.25] ^d^
Grandmother smoked when pregnant	PGM	Verbal IQ at 8	Boys	-2.51 [-2.36, -0.66] ^c^
	PGM	Performance IQ	All	-2.67 [-4.00, -1.34] ^a^
			Boys	-2.01 [-3.63, -0.39] ^d^
			Girls	-1.79 [-3.52, -0.06] ^d^

^a^P<0.0001; ^b^P<0.001; ^c^P<0.01; ^d^P<0.05. ^y^trend in IQ per standardised GDP per year. MGM = maternal grandmother; MGF = maternal grandfather; PGM = paternal grandmother; PGF = paternal grandfather.

### Total IQ at age 15

Results for the relationships between the characteristics of the grandparents and the grandchild’s Total IQ at age 15 are shown in *Extended data*, Tables S5–S12 ( Iles Caven *et al*., 2020). In summary these show that two possible transgenerational associations were retained in the model concerning the **maternal** line: the GDP trend level at maternal grandmother’s year of birth and whether the maternal grandfather was a smoker ( *Extended data*, Table S7) ( Iles Caven *et al*., 2020). When the grandsons were analysed separately, the trend in the maternal grandmother’s GDP trend level was retained, but not the grandfather’s smoking. For the granddaughters, whether the maternal grandfather was a smoker was retained but not the maternal grandmother’s GDP trend level at birth ( Table 7 and *Extended data*, Tables S8 and S9) ( Iles Caven *et al*., 2020).

Overall, for the **paternal** line, the only potential transgenerational association retained was whether the grandfather was a smoker ( *Extended data*, Table S8) ( Iles Caven *et al*., 2020). Separate analysis for grandsons resulted in no potential transgenerational associations remaining in the model ( *Extended data*, Table S11), but for the granddaughters both the GDP trend level in the paternal grandfather’s year of birth and whether he smoked were retained ( Table 7 and *Extended data*, Table S12) ( Iles Caven *et al*., 2020). 

### Verbal and performance IQs at age 8

The data concerning the verbal and performance subgroups of the total IQ measure were analysed in a similar way (see *Extended data*, Tables S13–S27) ( Iles Caven *et al*., 2020) and are summarised below.

***(a) Verbal IQ***

Unadjusted associations for the variables in the **maternal line** were apparent for all the variables considered with the exception of the business cycle at the year of birth, maternal parity and the ethnic group of the maternal grandfather ( *Extended data*, Tables S13 and S14) ( Iles Caven *et al*., 2020). Adjusted analysis revealed the same five variables being retained in the model as found for the Total IQ at 8 – none of these variables were potential transgenerational associations; similar results were found for grandsons and granddaughters when analysed separately ( *Extended data*, Tables S15, S17 and S18) ( Iles Caven *et al*., 2020).

For the **paternal line**, adjusted analysis of all grandchildren resulted in four variables being retained, one of which was a possible transgenerational association: GDP trend in year of birth of the paternal grandmother ( *Extended data*, Table S16) ( Iles Caven *et al*., 2020). There were different results for the grandsons (where the smoking of the paternal grandmother in the pregnancy resulting in the father was retained, but not the trend in GDP), and the granddaughters (with the trend in GDP as well as the business cycle prevailing in the year of birth of the paternal grandmother) ( Table 7 and *Extended data*, Tables S19 and S20) ( Iles Caven *et al*., 2020).

***(b) Performance IQ***

For the **maternal line**, all variables had unadjusted associations with the exception of the business cycles, ethnic groups and parity ( *Extended data*, Tables S21 and S22) ( Iles Caven *et al*., 2020). Upon adjustment, two potential transgenerational associations were retained – the trend in GDP with maternal grandmother’s year of birth and whether the maternal grandfather was a smoker. The same association with the paternal grandfather being a smoker was apparent for both grandsons and granddaughters, but the association with the trend in GDP of birth year of the maternal grandmother was only retained in the grandson’s model ( *Extended data*, Tables S24 and S25) ( Iles Caven *et al*., 2020).

Among the variables describing the **paternal line**, neither the paternal grandfather smoking nor any variables associated with GDP entered, but there was an association with the paternal grandmother smoking in the pregnancy that resulted in the birth of the study father ( *Extended data*, Table S23) ( Iles Caven *et al*., 2020). This exposure was retained in the model for the grandsons but not the granddaughters. For the granddaughters the trend in GDP when the paternal grandmother was born was retained ( *Extended data*, Tables S26 and S27) ( Iles Caven *et al*., 2020). 

## Discussion

**Figure 1.**
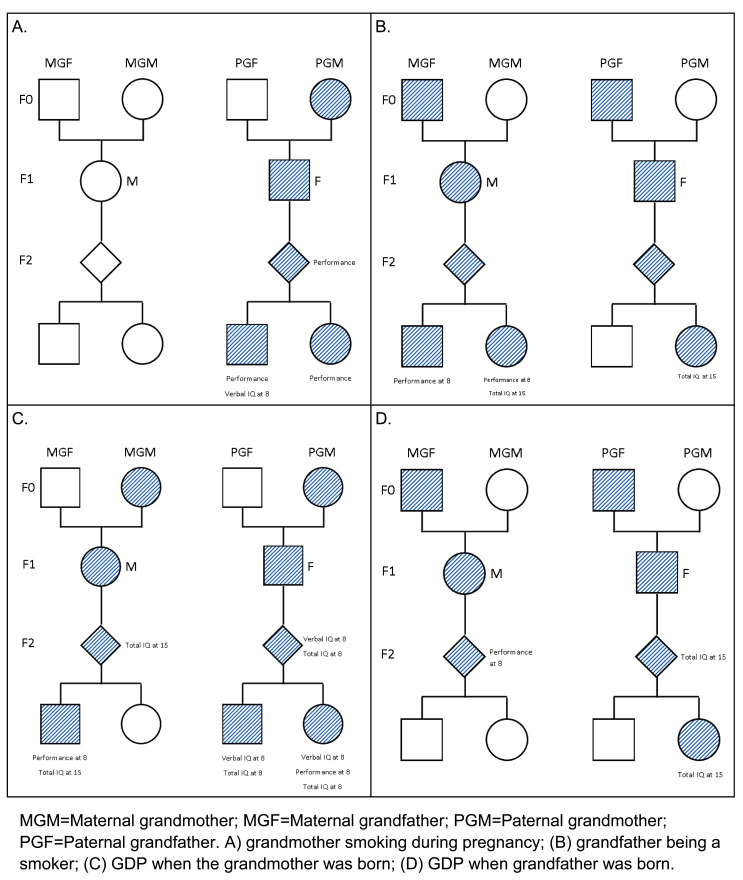
The adjusted associations at P<0.05 between grandparental exposures and grandchild IQ. ( **A**) Grandmother smoking during pregnancy. ( **B**) Grandfather being a smoker. ( **C**) GDP when the grandmother was born. ( **D**) GDP when grandfather was born. MGM=Maternal grandmother; MGF=Maternal grandfather; PGM=Paternal grandmother; PGF=Paternal grandfather.

 This paper follows on from our earlier studies showing differing associations between the grandmother smoking in pregnancy and the outcome of the grandchild, often depending on the grandchild’s sex. We previously demonstrated clear associations between grand-maternal prenatal smoking and intrauterine growth (including head circumference at birth); child growth and increasing body mass index (BMI) in adolescence; asthma; autism and autistic traits. We had, therefore, hypothesised that there would be associations between the grandmaternal smoking in pregnancy and the IQ of the grandchild. Unlike our previous studies, we decided here to include other exposures of the grandparents that might exhibit associations in later generations. We highlighted for consideration, among the variables collected, the smoking habit of each grandfather, and the GDP at the year of birth of each grandparent as potential exposures that might show transgenerational associations (this was analysed as a trend as well as a component of the business cycle). We used the social variables available concerning the grandparents as confounders since it was likely that the social circumstances and education would be passed from one generation to another by social patterning rather than by genetic or transgenerational non-genetic inheritance. Our research questions were largely hypothesis generating. We did not specify the direction in which associations might be found, but we did predict that associations were likely to be specific to the sex of the grandparent exposed and/or the sex of the affected grandchild. We found 25 significant associations with these variables: 9 down the female line, and 16 down the male line ( Figure 1 and Table 7). The results are summarised below.

### Grandmother smoking in the pregnancy resulting in the birth of the parent

We found no significant associations with a history of the maternal grandmother smoking when pregnant with the study mother. For the paternal grandmother smoking in the pregnancy resulting in the birth of the study father, there were associations with a lower Performance IQ for all 8-year-old grandchildren as well as for grandsons and granddaughters when analysed separately; there was also an association with a lower verbal IQ for the grandsons. There were no associations with the Total IQ at either age 8 or age 15 ( Figure 1A; Table 7).

### Grandparents’ smoking habit

There were no adjusted associations with the grandmother smoking, other than when she had smoked during pregnancy as described above. Grandfather’s smoking, however, was associated down the maternal line with Performance IQ for all grandchildren, grandsons only and granddaughters only, and with Total IQ at 15 with granddaughters only and with all grandchildren. Down the male line, grandfather smoking was associated with Total IQ at 15, especially in granddaughters rather than grandsons ( Figure 1B).

### Trends in GDP in the year of birth of the grandmothers and grandfathers

The trend in the GDP present during the year of birth of each grandparent showed different adjusted associations with the IQ outcomes: (a) that of the maternal grandmother was associated with the Total IQ at age 15 for all grandchildren and particularly for the grandsons; (b) that of the paternal grandmother was associated with the Total IQ at 8, and the verbal IQ for all grandchildren as well as for the grandsons and granddaughters considered separately – in addition the granddaughters (but not grandsons) had a lower performance IQ when the paternal grandmother had been born in a year with a higher GDP; (c) the trend in the GDP for the years of birth of the maternal grandfather was associated with the performance IQ among all grandchildren, but not statistically significant at P<0.05 among either the grandsons or granddaughters when analysed separately; (d) the trend among the offspring of the paternal grandfathers was associated with Total IQ at 15 for all grandchildren, and for granddaughters when analysed separately ( Table 7 and Figure 1C, D).

### GDP business cycle at birth of grandparents

Only one association with business cycle survived adjustment (positive association between paternal grandmother business cycle and verbal IQ of granddaughters) ( *Extended data*, Table S20) ( Iles Caven *et al*., 2020).

### Adjusting for multiple tests

The study was designed to develop hypotheses rather than to test them. We carried out a total of 12 tests for each of four exposures and four outcomes – i.e. 192 tests. Chance would dictate that there would be at least two results with a P value <0.01, and 0.2 at P<0.001. The current adjusted analyses identified 15 results at P<0.001, 13 of which had a P<0.0001. Thus, there are many more associations than would be expected by chance. However, that does not, at this stage, imply causal effects – even though some of the features were hypothesised (i.e. that the results would differ according to whether the maternal or paternal line, and whether grandson or granddaughter was considered).

It should be acknowledged that, although the numbers of individual grandparent/grandchild pairs were larger than in other studies, they are still smaller than would be required to demonstrate significance for any other than large effect sizes. Importantly, although the numbers considered down the paternal line were only about half of those considered down the female line, it was the paternal line that showed more significant associations.

### Could grandparental smoking have a transgenerational effect?

We have shown an association here between the paternal grandmother smoking in the pregnancy resulting in the father and performance and verbal IQ, especially in the grandsons. This mirrors a study that we had published earlier looking at fetal growth when the paternal grandmother and the study mother had both smoked in pregnancy - this showed a deficit in head circumference at birth among the grandsons, with an accompanying reduction in IQ at 8 years. In that study we controlled for social circumstances present at the time of birth of the grandchild ( Miller *et al.*, 2014a). Here we have controlled for social circumstances present at the time of birth and childhood of the grandparents. Both methods of analysis have shown an association between paternal (but not maternal) grandmother smoking prenatally and a deficit in IQ of the grandchildren, especially the grandsons. This was independent of whether the study mother had smoked or not (data not shown).

We have not examined possible effects of the **grandfather** smoking prior to the pregnancy, although we have shown associations between the **father** starting to smoke regularly in mid-childhood and his son’s obesity, which increased with age ( Golding *et al.*, 2019; Northstone *et al.*, 2014). Here if the grandfather was reported to be a smoker, we have assumed that he was likely to have smoked before the study parent was conceived, since past population survey data indicate that the commencement of smoking whilst a teenager was particularly high, and in the 1920s–1930s there is substantial evidence that the prevalence of smoking was approximately 90% of men in the UK ( Forey *et al.*, 2016). 

 In this study, we have shown associations between Total IQ at age 15 with both the maternal and paternal grandfathers being smokers. The effect sizes were larger for the granddaughters than the grandsons. There was also an association between the maternal grandfather being a smoker and performance IQ for both grandsons and granddaughters. There were no associations, however, with Total IQ at 8 or verbal IQ at 8.

### GDP in the year of birth of the grandparents

Of the 96 tests carried out, 13 demonstrated an association between increasing levels of GDP at the time of a grandparent’s birth and a reduction in the IQ of their grandchild. This was particularly true for the years of birth of the grandmothers as opposed to grandfathers (10 and 3 respectively). However, the trends in year of birth of the grandparents and the trends in GDP were strongly correlated ( *Extended data*, Table S28a, b) ( Iles Caven *et al*., 2020), and it is possible that the trend in GDP was measuring the same construct as the trend in year of birth. We did not force the two variables into the same regressions because of the collinearity that would result.

### Strengths and limitations of the study

Among the strengths are the following: (a) The data on the grandparents were collected at the time the study mother was pregnant, and therefore could not be biased by knowledge of the IQ of the offspring. (b) There was unlikely to be gross misreporting of the smoking habits of the grandparents – the parents were likely to know their parents’ smoking habits. (c) It is noteworthy that the grandmothers would mostly have been pregnant with the study parents long before the health message concerning the disadvantages to the unborn baby of maternal smoking were known. Consequently, it is unlikely there would be biases concerning those grandmothers who were health conscious and those who were not in regard to smoking. (d) Although the information concerning whether the grandmother (F0) smoked during the pregnancy resulting in the birth of the parent (F1) was sometimes missing, we have assumed she did smoke prenatally if she smoked but it was not known if this occurred during pregnancy. There is evidence that this was an appropriate assumption since the mean birthweights of the parents were reduced to the extent that one would expect if smoking had occurred ( Pembrey *et al.*, 2014). (e) The proportion of the offspring for whom a valid measure of IQ was available was reasonably consistent for the different grandparent measures ( Table 2).

There are several limitations: (i) There are no replication studies currently published to confirm (or refute) these findings. (ii) Information on the grandparents’ environments relied on reports from the parents – although these are unlikely to be 100% accurate, others have shown that parental report of their parents smoking in pregnancy has a high reliability (kappa = 0.61; Pape *et al.*, 2019). (iii) It could be argued that the factors controlled for in this study were inappropriate, or that key covariates were missing. We deliberately controlled for the social and demographic variables current at the time of the grandmother’s pregnancy or earlier, rather than social factors operating at the time of the birth of the grandchild, since there is evidence, for example, that prenatal smoke exposure is linked to a variety of poor outcomes which could influence the development and achievements of the subsequent generation (i.e. F1). Thus, controlling for F1 factors such as their education or social status might be an over-control. (iv) Selective fertility among F0 and F1 is an additional limitation of this study (as it is for any intergenerational study). For example, those in F0 exposed to adverse conditions might be less fertile and hence be less likely to generate any F2 offspring. This could lead to a bias in interpretation.

## Conclusions

In line with our other studies, we find that smoking of the grandparents is associated with demonstrable changes in the grandchild, this time involving IQ; and similar to our other studies, we found that the associations were stronger in one sex than the other. If substantiated in other studies this raises the question as to what the mechanism might be. IQ is a composite measure, combining a number of different features, and we hypothesise that there are likely to be different mechanisms in consequence. We have argued elsewhere ( Hall *et al*., 2020), ‘At present we are largely ignorant of the causal pathways underpinning this and similar intergenerational responses to grandmaternal smoking in pregnancy. As indicated in the Introduction, the exposure of either (F1) parent to tobacco as a fetus may have resulted in a direct xenobiotic exposure to both their developing somatic tissues and their emerging germline, ultimately destined for any (F2) grandchildren. Alternatively, it may be the generalised DNA damage caused by (F0) grandmaternal smoking and/or the consequent DNA damage response of the fetus that modifies the emerging germline and alters F2 embryonic brain development. Clearly very complex, it is premature to speculate further.’

## Data availability

### Underlying data

ALSPAC data access is through a system of managed open access. The steps below highlight how to apply for access to the data included in this data note and all other ALSPAC data:

1. Please read the ALSPAC access policy ( http://www.bristol.ac.uk/media-library/sites/alspac/documents/researchers/data-access/ALSPAC_Access_Policy.pdf) which describes the process of accessing the data and samples in detail, and outlines the costs associated with doing so.
2. You may also find it useful to browse our fully searchable research proposals database ( https://proposals.epi.bristol.ac.uk/?q=proposalSummaries), which lists all research projects that have been approved since April 2011.
3. Please submit your research proposal ( https://proposals.epi.bristol.ac.uk/) for consideration by the ALSPAC Executive Committee. You will receive a response within 10 working days to advise you whether your proposal has been approved.

### Extended data

Figshare: Supplementary data on grandparental smoking and environmental exposures and IQ in grandchildren.pdf. https://doi.org/10.6084/m9.figshare.12789359 ( Iles Caven *et al*., 2020).

File ‘Additional data.pdf’ contains Tables S1–S28.

Extended data are available under the terms of the Creative Commons Attribution 4.0 International license (CC-BY 4.0).
