# Supplementary material for: Grandchild’s IQ is associated with grandparental environments prior to the birth of the parents
Source: Wellcome Open Res. 2021 Mar 15;5:198. Originally published 2020 Aug 24. [Version 2] doi: 10.12688/wellcomeopenres.16205.2 (PMC8008356; doi:10.12688/wellcomeopenres.16205.2)
Supplement: Peer review history

# Peer review: Reviewer response for version 2

**DOI:** 10.21956/wellcomeopenres.18160.r43050

**Published:** 2021-03-26

**Authors:** Håkan Karlsson, Hugo Sjöqvist

**Affiliations:** 1 Department of Global Public Health, Karolinska Institutet, Stockholm, Sweden; 2 Department of Neuroscience, Karolinska Institutet, Stockholm, Sweden

The authors have responded to our comments and suggestions and we agree that you can change the status to “approved” on our part.

 Upon re-reading the manuscript, we noted these errors:

1. Table S10 has a point-estimate (-8.84) which is outside of its confidence interval (-4.99, -2.68).
2. In Table S26, there is a numerical error of “1-0.11” instead of “-0.11”.

To improve clarity, we would also like to encourage the authors to specify the linear regression method used (i.e., Ordinary Least Squares or Maximum Likelihood estimation). Our approval is however not conditional on a response to these remarks.

Is the work clearly and accurately presented and does it cite the current literature?

Partly

If applicable, is the statistical analysis and its interpretation appropriate?

Partly

Are all the source data underlying the results available to ensure full reproducibility?

Yes

Is the study design appropriate and is the work technically sound?

Yes

Are the conclusions drawn adequately supported by the results?

Yes

Are sufficient details of methods and analysis provided to allow replication by others?

Partly

Reviewer Expertise:

Statistics, Neuroscience.

We confirm that we have read this submission and believe that we have an appropriate level of expertise to confirm that it is of an acceptable scientific standard.

# Peer review: Reviewer response for version 2

**DOI:** 10.21956/wellcomeopenres.18160.r43051

**Published:** 2021-03-19

**Authors:** Cecilie Svanes

**Affiliations:** 1 Centre for International Health, Department of Global Public Health and Primary Care, University of Bergen, Bergen, Norway

No further comments, approved.

Is the work clearly and accurately presented and does it cite the current literature?

Yes

If applicable, is the statistical analysis and its interpretation appropriate?

I cannot comment. A qualified statistician is required.

Are all the source data underlying the results available to ensure full reproducibility?

Yes

Is the study design appropriate and is the work technically sound?

Yes

Are the conclusions drawn adequately supported by the results?

Yes

Are sufficient details of methods and analysis provided to allow replication by others?

Yes

Reviewer Expertise:

Multi-generation research in humans, mainly in asthma/allergies.

I confirm that I have read this submission and believe that I have an appropriate level of expertise to confirm that it is of an acceptable scientific standard.

# Peer review: Reviewer response for version 1

**DOI:** 10.21956/wellcomeopenres.17796.r40627

**Published:** 2020-10-22

**Authors:** Håkan Karlsson, Hugo Sjöqvist

**Affiliations:** 1 Department of Global Public Health, Karolinska Institutet, Stockholm, Sweden; 2 Department of Neuroscience, Karolinska Institutet, Stockholm, Sweden

In their paper, Golding and co-workers explore the potential associations between environmental exposures in grandparents (UK gross domestic product, GDP, at their time of birth and their smoking history) and performance of their grandchildren on tests of intelligence. The primary objective was to generate support for the very interesting hypothesis that such exposures have transgenerational, sex-specific, effects detectable at the level of cognitive performance. To this end the authors explore multi-generational data available in ALSPAC.

 In their introduction, the authors cite relevant literature including some of the many studies to date that have reported on the high, and increasing, degree of heritability of adult intelligence and the contribution of common genetic variation to this complex trait. The authors could also acknowledge the large degree of genetic correlation that has been reported between intelligence and many mental and somatic disorders and traits. Of relevance to the current study is the highly significant inverse genetic correlation between general cognitive ability and smoking history reported by Trampush *et al*. (Trampush, 2017) ^1^. Such data suggests that a phenotypic association between smoking and intelligence observed among related individuals is expected on a purely genetic basis. The authors could also elaborate a bit more on the rationale for choosing GDP as an exposure in the introduction.

 The authors used questionnaire data on exposures; grandparental smoking history (provided by parent) and the gross domestic product of the UK (GDP) at the time of birth of the grandparent along with the co-variates grandparental educational level, social class, birth-year, age at birth of parent (all provided by parents). The authors suggest that associations with grandparental smoking history and/or GDP are likely to represent inter/transgenerational effects and investigate these using multiple stepwise regression models including the co-variates. We suggest that the authors mention what type of regression model they are using along with a clarification of the inconsistent reporting of p-values, exact vs. e.g. <0.001. While the authors use a continuous outcome, there is no information on the nature of a possibly non-linear relationship between GDP and IQ, which contradicts the authors’ aim to “determine the relationships between different aspects of the environment of the grandparents (F0) of the study children (F2)”.

 We would also like the authors to explain their statement “taking care to ensure that we avoided Type I errors as much as possible”, in more detail. 

 It has been reported that the heritability of smoking habits varies between men and women and between environments that are more or less permissive to smoking (Li, 2003) ^2^. We would therefore like the authors to provide a more justifiable rationale than “hypothesis generation” for omitting parental co-variates from the current study, pending that such data is available.

 The discussion would benefit from less repetition of the results section and more of actual discussion of the potential mechanisms underlying the results and suggestions for future studies of this complex issue.

Is the work clearly and accurately presented and does it cite the current literature?

Partly

If applicable, is the statistical analysis and its interpretation appropriate?

Partly

Are all the source data underlying the results available to ensure full reproducibility?

Yes

Is the study design appropriate and is the work technically sound?

Yes

Are the conclusions drawn adequately supported by the results?

Yes

Are sufficient details of methods and analysis provided to allow replication by others?

Partly

Reviewer Expertise:

Statistics, Neuroscience.

We confirm that we have read this submission and believe that we have an appropriate level of expertise to confirm that it is of an acceptable scientific standard, however we have significant reservations, as outlined above.

## References

[1] : GWAS meta-analysis reveals novel loci and genetic correlates for general cognitive function: a report from the COGENT consortium. *Mol Psychiatry*.22(3) : 10.1038/mp.2016.244 336-345 10.1038/mp.2016.244 28093568PMC5322272

[2] : A meta-analysis of estimated genetic and environmental effects on smoking behavior in male and female adult twins. *Addiction*.2003;98(1) : 10.1046/j.1360-0443.2003.00295.x 23-31 10.1046/j.1360-0443.2003.00295.x 12492752

# Response

**Published:** 2020-12-14

**Authors:** Jean Golding

**Affiliations:** University of Bristol, UK

We are grateful to the reviewers for their comments. Our responses are below.

1. They suggest that the correlation between intelligence and many different disorders such as those indicated by Trampush and colleagues (2017) indicates that one would expect an association between smoking and intelligence purely on genetic grounds. If this were an explanation of our findings, we would surely expect similar associations between paternal grandparents’ smoking habits with IQ and those between maternal grandparents’ smoking habits and IQ.
2. We agree that the discussion of the use of GDP as an exposure should be expanded, and this we have now done at the end of the second paragraph of the Introduction.
3. We have expanded the description of the statistical methods used to encompass: (i) our use of stepwise multiple regression in order to disentangle the collinear factors that are independently associated with each outcome. (ii)  In regard to the possible non-linear nature of a GDP effect, the additional factor ‘business cycle’ identified the years where the GDP did not fall within a linear trajectory and was included along with the variable indicating the trend in GDP. (iii) we have expanded the sentence on avoiding Type I errors to include the reason for not taking account of multiple testing.
4. The issue of how the P values are presented is noted. Different methods are used, but we argue that the meaning is clear throughout. We will not change on this occasion, but take note for further manuscripts.
5. We have added some suggestions as to mechanisms at the end of the paper.

Alessie, R. J., Angelini, V., van den Berg, G. J., Mierau, J. O., & Viluma, L. (2019). Economic conditions at birth and cardiovascular disease risk in adulthood: Evidence from post-1950 cohorts. *Social Science & Medicine*, *224*, 77-84.

 van den Berg, G. J., Paul, A., & Reinhold, S. (2020). Economic conditions and the health of newborns: Evidence from comprehensive register data. *Labour Economics*, *63*, 101795.

# Peer review: Reviewer response for version 1

**DOI:** 10.21956/wellcomeopenres.17796.r40140

**Published:** 2020-09-23

**Authors:** Cecilie Svanes

**Affiliations:** 1 Centre for International Health, Department of Global Public Health and Primary Care, University of Bergen, Bergen, Norway

This paper presents an important hypothesis-generating analysis of a highly relevant topic, addressing potential associations of grandparental environmental factors (Gross Domestic Product measures, grandparental smoking) with measures of IQ, with focus on sex-specific patterns. Overall, the work is well presented, the authors guide the reader very well through complex issues, and the conclusions are appropriately cautious but still very valuable.

 The paper cites relevant literature related to the outcome IQ as far as I can appreciate, but limits literature related to the exposures mainly to publications from their own study. I suggest to add a reference validating offspring’s reporting of parental smoking (Pape K *et al*. 2019 ^1^).

 About the statistical methods, the argument that correction for multiple testing is not done in this hypothesis generating analysis is sound. The assumptions underlying the analyses are clearly presented to the reader. I am not convinced about the appropriateness of using a backwards step-wise approach but on the other hand acknowledge the complexity of these analyses; I suggest that a statistician with some experience with multi-generation analyses comments on this. For the discussion, I would have liked more speculation with regard to potential underlying biological mechanisms.

   

 Considering the terminology, the authors state that they will use the term “transgenerational” whether inter- or transgenerational mechanisms are assumed. One may hope that the field is becoming somewhat more familiar to readers and that one may distinguish between these in near future. Two details as to the exposure description, (i) with the term “smoking history” I would expect more than a question on whether a parent smoked, and suggest to merely give the wording of the question; ii) last sentence “This assumption was validated…” Showing lower birthweight in mothers reported to have smoked hardly “validates” exposure during pregnancy, this should be worded more carefully.

Is the work clearly and accurately presented and does it cite the current literature?

Yes

If applicable, is the statistical analysis and its interpretation appropriate?

I cannot comment. A qualified statistician is required.

Are all the source data underlying the results available to ensure full reproducibility?

Yes

Is the study design appropriate and is the work technically sound?

Yes

Are the conclusions drawn adequately supported by the results?

Yes

Are sufficient details of methods and analysis provided to allow replication by others?

Yes

Reviewer Expertise:

Multi-generation research in humans, mainly in asthma/allergies.

I confirm that I have read this submission and believe that I have an appropriate level of expertise to confirm that it is of an acceptable scientific standard.

## References

[1] : Agreement of offspring-reported parental smoking status: the RHINESSA generation study. *BMC Public Health*.2019;19(1) : 10.1186/s12889-019-6414-0 94 10.1186/s12889-019-6414-0 30665381PMC6341700

# Response

**Published:** 2020-12-14

**Authors:** Jean Golding

**Affiliations:** University of Bristol, UK

Thank you for the Pape reference – We were not aware of it until now, and have now referred to it in this paper.

 You ask for more discussion about possible mechanisms. We have added a section at the end of the Conclusions that answers this.

 You also ask for the actual wording of the smoking in pregnancy question – and this is now included in the Methodology section.

 You pointed out that showing that exposed mothers had lower birthweight was not, strictly speaking, a validation. Rather, as we now state more accurately, it provides face validity to the claim.
